# Hump Nose and Coexisting Deviation in Rhinoplasty: Their Association and Surgical Considerations

**DOI:** 10.3390/jcm15010158

**Published:** 2025-12-25

**Authors:** Su Jin Kim, Je Ho Bang, Kun Hee Lee

**Affiliations:** Department of Otorhinolaryngology-Head and Neck Surgery, Kyung Hee University Hospital at Gangdong, Kyung Hee University College of Medicine, Seoul 05278, Republic of Korea; sujin.kim@khu.ac.kr (S.J.K.); jehosuk@naver.com (J.H.B.)

**Keywords:** hump, nasal deviation, deviated nose, rhinoplasty, osteotomy, camouflage

## Abstract

**Background/Objectives**: Dorsal humps and nasal deviation are common deformities in rhinoplasty. While existing classification systems characterize these deformities individually, their concurrent presentation complicates surgical planning. The aim of this study was to examine the association between dorsal humps and nasal deviation in rhinoplasty patients and assess their influence on surgical decision-making. **Methods**: This retrospective study analyzed 90 consecutive patients who underwent primary rhinoplasty between January 2020 and December 2023. Dorsal humps were classified as isolated or generalized. Nasal deviation was initially categorized into five types, then regrouped by bony involvement: bony deviation versus cartilaginous only deviation. The association between humps and deviations was examined, and their impact on surgical techniques was analyzed. **Results**: Dorsal humps were present in 64 (71%) and deviations in 63 (70%) patients. A significant association was observed between hump presence and deviation (*p* < 0.001); 83% of patients with humps exhibited concurrent deviation compared to 39% in those without humps. Patients with deviations showed 7.7-fold increased odds of having a concurrent hump (*p* < 0.001). Bony deviation demonstrated a particularly strong association with hump presence (OR 14, *p* < 0.001), while cartilaginous only deviation showed no significant association. Binary logistic regression identified deviation presence as the primary predictor for requirements of both osteotomy (OR 3.5, *p* = 0.025) and camouflage (OR 4.4, *p* = 0.025). **Conclusions**: Dorsal humps and nasal deviation frequently coexist, particularly humps with bony deviations. Surgical decision-making is more strongly influenced by deviation status than by hump presence. An integrated classification system incorporating both characteristics is needed to optimize surgical planning.

## 1. Introduction

Dorsal hump deformities are a common concern in rhinoplasty, and their classification aids surgical planning and communication [1]. Traditional hump classification systems have focused primarily on surface contour morphology, categorizing deformities as isolated, generalized, and relative based on the extent and distribution of dorsal convexity [1]. In Caucasian patients, these humps typically present as prominent osseocartilaginous protrusions [2], while Asian patients often exhibit smaller humps associated with characteristic features such as low radix and under projected nasal tips [3,4]. Recently, preservation rhinoplasty approaches have incorporated factors such as the anticipated amount of reduction, nasal bone shape, and hump composition into their classification strategies [5].

Nasal deviation, an asymmetry of the nasal pyramid from the facial midline, is a significant challenge in rhinoplasty due to its complex nature and combined aesthetic and functional implications [6,7]. Various classification systems categorize these diverse deformities to facilitate surgical planning. One widely recognized system classifies deviations into five types based on the orientation of the bony pyramid and cartilaginous vault relative to the facial midline [8]. Other classifications describe deviations using morphological terms like C-shaped, reverse C-shaped, I-shaped, and S-shaped deformities, reflecting specific curvatures of the septal or external nasal axis [9]. These systems aim to streamline diagnosis, enabling more precise surgical strategies and individualized treatment plans [10].

However, while these classification systems effectively characterize individual deformities, they evaluate hump and deviation as separate entities despite frequent observations of their co-occurrence. This independent assessment approach may not adequately reflect the morphological complexity encountered in clinical practice, where both deformities often present simultaneously and may influence surgical planning collectively rather than independently.

Although dorsal humps and nasal deviation are among the most common deformities encountered in rhinoplasty patients, previous studies have primarily reported their individual prevalence within surgical cohorts. One study documented deviation in 39% of Asian patients with dorsal humps [3], while others have reported the separate prevalence of each deformity without analyzing their co-occurrence [11]. These investigations provided descriptive data without examining the quantitative association between these deformities or how their presence influences surgical decision-making.

To our knowledge, no study has tested whether dorsal humps and deviation patterns are statistically associated, nor explored whether their co-occurrence influences the requirement for surgical techniques such as osteotomy. As a result, surgeons often rely on clinical impressions that these deformities frequently coexist, but objective data quantifying their association and its relevance to operative planning remain limited. Therefore, this study aimed to examine the quantitative association between dorsal humps and nasal deviation and to assess their influence on surgical technique requirements in primary rhinoplasty.

## 2. Materials and Methods

### 2.1. Study Design and Patients

This retrospective study analyzed patients who underwent primary rhinoplasty at a university hospital between January 2020 and December 2023. The study was approved by the Institutional Review Board and informed consent requirements were waived due to the retrospective design.

We retrospectively reviewed medical records and standardized preoperative photographs of 100 consecutive patients who underwent primary rhinoplasty. All procedures were performed by a single surgeon (K.H.L.) to ensure consistency in surgical decision-making. Inclusion criteria were: (1) patients aged 18 years or older; (2) primary rhinoplasty; and (3) availability of complete standardized preoperative photographs (frontal, lateral, oblique, and basal views). Exclusion criteria were: (1) previous rhinoplasty; (2) known congenital craniofacial anomalies affecting nasal morphology; (3) a documented history of significant nasal or facial trauma; (4) pseudohump misclassification; and (5) incomplete clinical or photographic documentation. Based on clinical records, 100 patients met the initial eligibility criteria, and an additional 10 were excluded during photographic review, resulting in a final cohort of 90 patients.

### 2.2. Morphological Assessment

As part of routine clinical evaluation for patients undergoing rhinoplasty, preoperative CT scans were reviewed to assess overall nasal anatomy, including the bony pyramid, septum, and cartilaginous framework. However, CT scans were not re-reviewed or used for quantitative measurements for research purposes, as the present analysis focused on external morphological characteristics assessed through standardized photographs and intraoperative findings.

Dorsal humps were evaluated using standardized lateral-view preoperative photographs by two independent authors (K.H.L. and S.J.K.). Intraoperative findings were also reviewed and incorporated into the final classification. Humps were categorized as either isolated or generalized based on the shape and extent of the dorsal convexity. An isolated hump was defined as a focal protrusion centered at the rhinion that disrupted an otherwise smooth dorsal profile, whereas a generalized hump involved a broad convexity spanning both the bony and cartilaginous vaults. Patients with pseudo humps—cases in which an apparent dorsal convexity was judged to result from a low radix or insufficient tip projection rather than from true dorsal fullness—were excluded from analysis.

Nasal deviation was evaluated by two independent authors based on standardized preoperative photographs and intraoperative findings were also incorporated into the classification. Deviation was initially defined according to a previously established system that classifies deviations into five types (I–V) based on the orientation of the bony pyramid and cartilaginous vault relative to the facial midline [8]. For the analysis, deviation was dichotomized a priori based on surgical relevance: bony deviation (Types I, II, and V), for which osteotomy is generally indicated, versus cartilaginous only deviation (Types III and IV), which are typically managed without bony intervention.

All morphological assessments were conducted by two experienced surgeons using standardized evaluation protocols to minimize observer bias and ensure reproducible classifications. To enhance consistency, both surgeons independently reviewed hump morphology using predefined criteria. In cases of disagreement, consensus was reached through discussion, with intraoperative findings serving as a reference when applicable.

### 2.3. Surgical Procedures and Data Collection

Operative records documented surgical approaches and interventions performed. We reviewed the choice between endonasal and external approaches, the requirements for osteotomy procedures including medial, lateral, percutaneous, or one-unit techniques, spreader graft placement, and camouflage grafting methods, with direction documented when applicable for each procedure.

### 2.4. Statistical Analysis

Data analysis was performed using R version 4.5.2 (R Foundation for Statistical Computing, Vienna, Austria). Descriptive statistics were presented as frequencies and percentages for categorical variables. Chi-square tests were used to evaluate associations between morphological features (hump presence, deviation presence and type) and surgical procedures. Fisher’s exact test was applied when expected cell counts were less than 5. Binary logistic regression analysis was performed to identify predictive factors for specific surgical interventions, with results presented as odds ratios (OR) with 95% confidence intervals. Statistical significance was set at *p* < 0.05.

## 3. Results

### 3.1. Patient Demographics and Morphological Features

The study included 90 patients with a mean age of 30 ± 12 years (range 18–72 years). The cohort comprised 65 males (72%) and 25 females (28%). Dorsal humps were present in 64 patients (71%), with isolated hump being more prevalent than generalized hump (61% vs. 39%, respectively). Deviation was present in 63 patients (70%), with the majority exhibiting bony involvement (84%) compared to cartilaginous only deviation (16%) (Table 1).

### 3.2. Association Between Hump and Deviation

**Figure 1:**
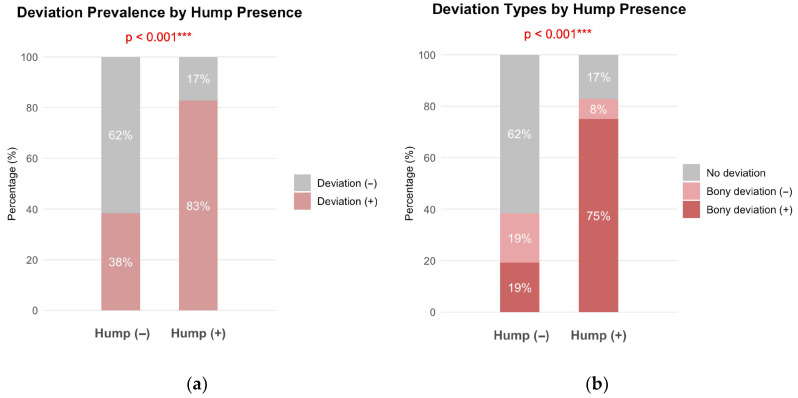
Association between dorsal hump presence and nasal deviation in primary rhinoplasty (n = 90). (**a**) Prevalence of nasal deviation in patients with a hump (n = 64) and without a hump (n = 26). (**b**) Distribution of deviation type according to hump presence. Deviation was classified by bony pyramid involvement as bony deviation versus cartilaginous only deviation. Numbers represent percentages within each group. Chi-square test, *** *p* < 0.001 for both comparisons.

A significant association was observed between hump presence and deviation (*p* < 0.001). Among patients without hump, 38% had concurrent deviation, whereas 83% of patients with hump exhibited accompanying deviation (Figure 1a). When stratified by deviation type, patients with hump showed a significantly higher prevalence of bony deviation compared to those without humps (75% vs. 19%, *p* < 0.001) (Figure 1b).

**Figure 2:**
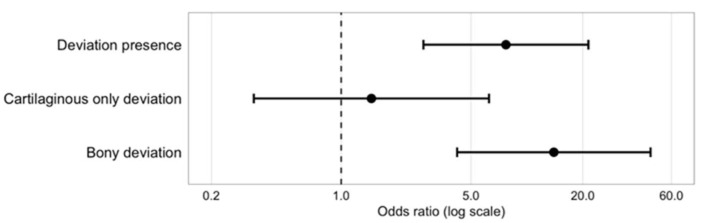
Odds ratios for the association between dorsal hump presence and deviation patterns. Odds ratios with corresponding 95% confidence intervals are shown for deviation presence, cartilaginous only deviation, and bony deviation, using patients without deviation as the reference group. Values greater than 1 indicate increased odds of hump presence.

Cross-tabulation analysis revealed that patients with deviation had 7.7-fold increased odds of having a concurrent hump compared to those without deviation (95% CI: 2.8–21, *p* < 0.001). When stratified by deviation type, patients with bony deviation showed 14-fold increased odds of hump presence (95% CI: 4.2–46, *p* < 0.001), while cartilaginous only deviation showed no significant association with hump presence (OR 1.5, 95% CI: 0.34–6.3, *p* = 0.62) (Table 2). The corresponding odds ratios for these associations are presented in Figure 2.

### 3.3. Surgical Procedure Patterns

Surgical approach selection showed higher rates of open approach in both patients with hump and patients with deviation, although these differences were not statistically significant (*p* = 0.60 and *p* = 0.16, respectively).

**Figure 3:**
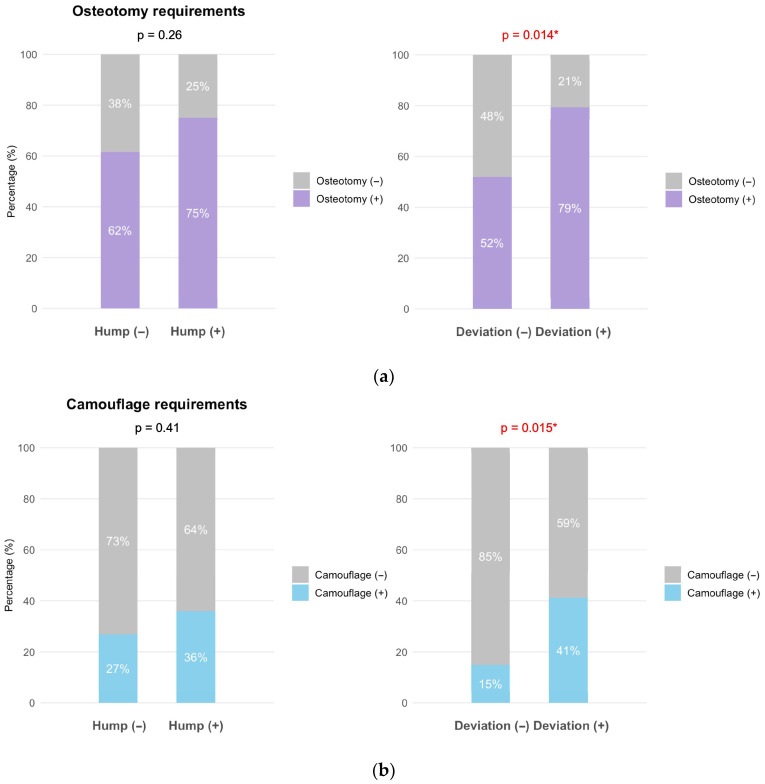
Impact of dorsal hump and nasal deviation on surgical decision-making in primary rhinoplasty (n = 90). (**a**) Osteotomy requirements by hump presence (**left**) and deviation presence (**right**). (**b**) Camouflage graft requirements by hump presence (**left**) and deviation presence (**right**). Patients with a hump (n = 64) and without a hump (n = 26), and those with deviation (n = 63) and without deviation (n = 27), were compared. Deviation was classified according to bony pyramid involvement as bony deviation versus cartilaginous only deviation. Numbers represent percentages within each group. Chi-square test with Monte Carlo simulation, * *p* < 0.05.

Osteotomy was more frequently performed in patients with hump than in those without hump (75% vs. 62%), but this difference was not statistically significant (*p* = 0.26). In contrast, osteotomy was significantly more frequently required in patients with deviation than in those without deviation (79% vs. 52%, *p* = 0.014) (Figure 3a).

The use of medial osteotomy did not differ significantly between patients with and without hump (*p* = 0.97). However, medial osteotomies were more commonly required in patients with deviation than in those without deviation (*p* = 0.029). Lateral osteotomies were more frequently required in both patients with hump (*p* = 0.045) and patients with deviation (*p* = 0.006).

Hump presence showed no significant association with camouflage procedure use (36% vs. 27%, *p* = 0.41). In contrast, camouflage procedures were more frequently required in patients with deviation than in those without deviation (41% vs. 15%, *p* = 0.015) (Figure 3b).

### 3.4. Predictors of Surgical Decision-Making

Binary logistic regression analysis identified deviation presence as the primary predictor of requirements for both osteotomy (OR 3.5, 95% CI: 1.2–10, *p* = 0.025) and camouflage (OR 4.4, 95% CI: 1.2–16, *p* = 0.025). Conversely, hump presence was not a significant predictor of either osteotomy requirements (OR 1.1, 95% CI: 0.35–3.3, *p* = 0.89) or camouflage requirements (OR 0.84, 95% CI: 0.26–2.7, *p* = 0.77) (Table 3).

## 4. Discussion

This study demonstrates a significant association between dorsal humps and nasal deviation, indicating that these features should be considered together in surgical planning. Current hump classification systems evaluate the dorsal contour without considering accompanying deviation [1], yet our findings reveal a markedly higher prevalence of deviation—particularly bony deviation—among patients with hump. Additionally, deviation status proved to be a stronger determinant of surgical maneuvers, particularly osteotomy and camouflage grafting. However, hump correction remains essential for achieving a harmonious dorsal line, and it must be planned in relation to the underlying deviation.

In our cohort, deviation was present in 83% of patients with hump, compared to 39% in those without. Notably, bony deviation showed a particularly strong association with hump presence (OR 14, *p* < 0.001). These findings suggest a structural association between dorsal convexity and underlying asymmetry. Although developmental factors have been proposed in prior literature as a potential basis for this coexistence, such mechanisms were beyond the scope of our study and should be regarded as hypothetical. Our results therefore indicate a phenotypic association rather than any specific embryologic or genetic linkage.

While developmental factors may provide a universal framework for the co-occurrence of hump and deviation, this association varies significantly across ethnicities. Studies examining nasal anatomy across different populations reveal substantial differences in baseline characteristics that influence the co-occurrence of these deformities [12,13]. Middle Eastern populations demonstrate particularly high concurrent rates, with dorsal hump prevalence of 85% and nasal asymmetries in 82% of rhinoplasty patients [14]. The structural basis for hump formation also differs across ethnicities: Middle Eastern patients show 70% of hump length with underlying nasal bones compared to 33.5% in East Asian populations [13]. Caucasian Mediterranean patients exhibit intermediate patterns with 78% dorsal hump prevalence [15]. This ethnic variation in both baseline prevalence and underlying structural characteristics suggests that population-specific anatomical factors may influence the co-occurrence patterns of hump and deviation.

Beyond genetic predisposition and ethnic anatomical variation, nasal trauma represents a significant acquired cause of concurrent hump and deviation [16,17]. Inadequate healing of nasal bone fractures may lead to fibrous union or subperiosteal bone remodeling, potentially resulting in lasting dorsal irregularities or secondary hump formation [18]. Simultaneously, if the fractured cartilaginous septum fails to stabilize in a neutral position, persistent deviation may occur due to tractional forces [17,18]. Moreover, trauma-induced depression of the lower third, including the septal cartilage, can lead to a relative prominence of the bony dorsum [17,18]. Although our study, being a retrospective review, may not fully ascertain all instances of trauma history, the high co-occurrence of hump and deviation observed may reflect the long-term effects of subclinical or unrecognized trauma.

Dorsal convexity has been classified into generalized, isolated, and relative hump types based on profile morphology and tip projection to guide surgical approach in Asian rhinoplasty [1]. While this framework provides visual categorization, it primarily reflects surface contour and does not address accompanying skeletal features such as deviation. A separate effort to standardize dorsal deformity assessment incorporated multiple parameters—including width, curvature, deviation, and projection—for both bony and cartilaginous structures [19], yet this approach assessed hump and deviation independently without examining their association. Deviation has been frequently observed in patients undergoing hump correction, with one study reporting a co-occurrence rate of 39% in Asian patients [3]. A more recent study identified hump and deviation as the two most prevalent nasal deformities among rhinoplasty patients, with reported rates of 59.0% and 54.6%, respectively, in that patient population [11]. Despite their frequent coexistence, the association between them has rarely been assessed quantitatively.

Beyond their shared etiology, the clinical significance of this association becomes evident in surgical planning. Our study demonstrates that surgical decision-making is more strongly influenced by deviation status than by hump presence. Medial osteotomy was performed more frequently in patients with deviation (*p* = 0.029), while hump presence showed no significant association (*p* = 0.97). This is probably because hump resection or rasping naturally creates an open roof, often eliminating the need for separate medial osteotomy [18]. Conversely, deviation correction frequently requires medial osteotomy for bony pyramid realignment [8,17]. Lateral osteotomy was necessary significantly more often in both hump and deviation cases (*p* = 0.045 and *p* = 0.006, respectively), with deviation showing stronger association. This aligns with established reports emphasizing lateral wall mobilization as essential for deviated nose correction [8,18]. Although deviation more strongly predicts the need for osteotomy, hump correction can also influence operative sequencing, as dorsal refinement frequently interacts with the steps required for deviation realignment.

Our decision to dichotomize the five-type deviation classification [8] into bony versus cartilaginous only categories was based on the surgical relevance of bony pyramid involvement. In the original framework, Types I, II, and V involve bony pyramid deviation and are typically managed with osteotomy, whereas Types III and IV represent cartilaginous only deformities that are often addressed without bony work. Because osteotomy planning largely depends on whether the bony pyramid is involved, this distinction provides a practical framework for interpreting deviation patterns. Our findings validate this dichotomization, as bony deviation showed a markedly stronger association with hump presence (OR 14, *p* < 0.001) compared to cartilaginous only deviation (OR 1.5, *p* = 0.62), supporting the clinical relevance of this simplified categorization.

Camouflage procedures were more frequently required in patients with deviation (*p* = 0.015) but showed no significant association with hump presence (*p* = 0.41). This reflects the need for secondary contouring to correct residual irregularities that may persist after realignment of the bony framework. The complexity of deviation correction often results in subtle contour irregularities that require refinement techniques, particularly in patients with thin nasal skin where minor irregularities become more apparent [20,21]. Although hump presence itself did not predict camouflage use in our cohort, camouflage techniques remain relevant in Asian rhinoplasty, where a small dorsal hump combined with a low radix or limited tip projection is often managed through selective radix or tip augmentation rather than relying solely on hump reduction [4,22].

Given the high co-occurrence of hump and deviation in our cohort, surgical planning may benefit from considering maneuvers that address both deformities within the same operative sequence. Oblique hump resection has been reported as a method that directs dorsal reduction toward the deviated side, allowing hump correction to contribute to bony realignment without requiring complete septal reconstruction [23]. Similarly, differential hump reduction guided by preoperative deviation measurements has been described, enabling asymmetric dorsal removal that supports restoration of nasal bone symmetry [24].

Current hump classification systems evaluate dorsal contour morphology independently [1], despite our evidence that hump and deviation frequently coexist. An integrated classification system that simultaneously considers both hump characteristics and deviation patterns could enhance surgical efficiency by enabling coordinated correction techniques that minimize residual deformities and reduce the risk of undercorrection [23,24]. Such a system would facilitate the development of standardized surgical algorithms that address both components systematically, potentially improving outcomes and reducing the need for revision procedures. However, these implications should be interpreted with caution, as they reflect population-specific patterns in our Asian cohort and may not be directly applicable to other groups without further validation.

This study represents, to our knowledge, the first comprehensive analysis to examine the association between hump and deviation within a single cohort. This addresses a gap in the literature where these deformities have primarily been studied separately. Morphological assessment by experienced surgeons combined with statistical validation provides objective evidence for associations previously recognized mainly in clinical practice. Additionally, the single-surgeon approach helped ensure consistency in surgical decision-making and technique selection by minimizing inter-surgeon variability.

Several limitations warrant consideration. First, the retrospective design limits our ability to establish causality between morphological features and surgical outcomes. This design also limits the ability to fully exclude unrecognized or undocumented trauma, which may have contributed to some of the observed morphological patterns. Second, the relatively small sample size from a single institution may limit the generalizability of our findings. Third, our patient population consisted entirely of Asian individuals, and anatomical variations across different ethnic groups may influence the applicability of these results to other populations. Fourth, morphology-based assessments inherently involve some degree of subjectivity, as formal inter-rater reliability metrics and quantitative criteria for pseudo hump exclusion were not applied. Although independent assessments by experienced surgeons and intraoperative confirmation were used to promote consistency, residual observer variability cannot be completely excluded. Future research addressing these limitations would help validate an integrated hump-deviation classification system and establish evidence-based surgical algorithms.

## 5. Conclusions

This study demonstrates a significant association between dorsal humps and nasal deviation, with the majority of patients with hump exhibiting concurrent deviation, particularly bony deviation. While surgical decision-making was more strongly influenced by deviation status than hump presence, the high co-occurrence rate necessitates integrated surgical approaches that address both deformities simultaneously. Current hump classification systems that evaluate dorsal morphology independently may inadequately capture this complexity. An integrated classification system incorporating both hump and deviation characteristics is needed to optimize surgical outcomes. The clinical relevance of these findings should be interpreted within the context of our Asian cohort, and further studies in more diverse populations will be needed to determine their broader applicability.

## Figures and Tables

**Table 1:** Patient Demographics and Morphological Features.

Characteristic	Value
Total patients, n	90
Age, years	
Mean ± SD	30 ± 12
Range	18–72
Sex, n (%)	
Male	65 (72)
Female	25 (28)
Hump presence, n (%)	64 (71)
Isolated *	39 (61)
Generalized *	25 (39)
Deviation presence, n (%)	63 (70)
With bony component **	53 (84)
Cartilaginous only **	10 (16)

SD, standard deviation. * Percentages calculated among patients with hump (n = 64). ** Percentages calculated among patients with deviation (n = 63).

**Table 2:** Cross-tabulation Analysis of Hump and Deviation with Odds Ratios.

Variable	Total n (%)	Hump Presence n (%)	OR (95% CI) *	*p* Value
Deviation presence				
No deviation	27 (30)	11 (41)	Reference	-
Deviation presence	63 (70)	53 (84)	7.7 (2.8–21)	<0.001 ***
Deviation type				
No deviation	27 (30)	11 (41)	Reference	-
Cartilaginous only	10 (11)	5 (50)	1.5 (0.34–6.3)	0.62
Bony deviation	53 (59)	48 (91)	14 (4.2–46)	<0.001 ***

* OR, odds ratio for hump presence; CI, confidence interval. *p* values from individual logistic regression analyses. *** *p* < 0.001.

**Table 3:** Multivariable Predictors of Surgical Requirements.

Variable	Osteotomy Requirements OR (95% CI)	*p* Value	Camouflage Requirements OR (95% CI)	*p* Value
Hump presence	1.1 (0.35–3.3)	0.89	0.84 (0.26–2.7)	0.77
Deviation presence	3.5 (1.2–10)	0.025 *	4.4 (1.2–16)	0.025 *

OR, odds ratio; CI, confidence interval. * *p* < 0.05. Multivariable logistic regression adjusted for hump and deviation presence.

## Data Availability

The data presented in this study are available on request from the corresponding author. The data are not publicly available due to privacy or ethical concerns.
